# Rapid determination of tricarboxylic acid cycle enzyme activities in biological samples

**DOI:** 10.1186/1471-2091-11-5

**Published:** 2010-01-28

**Authors:** Sergio Goncalves, Vincent Paupe, Emmanuel P Dassa, Jean-Jacques Brière, Judith Favier, Anne-Paule Gimenez-Roqueplo, Paule Bénit, Pierre Rustin

**Affiliations:** 1 Inserm, U676, Paris, 75019 France; 2 Université Paris 7, Faculté de médecine Denis Diderot, IFR02, Paris, France; 3 Inserm, U970, Paris, 75015 France; 4 Université Paris Descartes, Faculté de Médecine, Paris, France; 5 AP-HP, Hôpital Européen Georges Pompidou, Département de Génétique, Paris, France

## Abstract

**Background:**

In the last ten years, deficiencies in tricarboxylic acid cycle (TCAC) enzymes have been shown to cause a wide spectrum of human diseases, including malignancies and neurological and cardiac diseases. A prerequisite to the identification of disease-causing TCAC enzyme deficiencies is the availability of effective enzyme assays.

**Results:**

We developed three assays that measure the full set of TCAC enzymes. One assay relies on the sequential addition of reagents to measure succinyl-CoA ligase activity, followed by succinate dehydrogenase, fumarase and, finally, malate dehydrogenase. Another assay measures the activity of α-ketoglutarate dehydrogenase followed by aconitase and isocitrate dehydrogenase. The remaining assay measures citrate synthase activity using a standard procedure. We used these assays successfully on extracts of small numbers of human cells displaying various severe or partial TCAC deficiencies and on frozen heart homogenates from heterozygous mice harboring an SDHB gene deletion.

**Conclusion:**

This set of assays is rapid and simple to use and can immediately detect even partial defects, as the activity of each enzyme can be readily compared with one or more other activities measured in the same sample.

## Background

Interest in assaying tricarboxylic acid cycle (TCAC) enzyme activities has been rekindled by evidence that deficiencies in these enzymes cause a variety of human diseases [1,2], in contradiction to the long-held belief that any TCAC enzyme deficiency is lethal [3]. Several acquired conditions are characterized not only by post-translational alterations in electron transport respiratory chain proteins and impairments in mitochondrial calcium handling, but also by abnormalities in TCAC enzymes. Examples include heart failure in humans [4] and stress-related heart dysfunction induced in rats by chronic restraint [5]. Several inherited diseases have been ascribed to primary TCAC enzyme deficiencies (Table 1). For instance, primary succinate dehydrogenase (SDH; EC.1.3.99.1) deficiency results either in tissue degeneration with devastating early-onset encephalomyopathy or in tissue proliferation with formation of paragangliomas or other tumors [2]. Similarly, a mutation in the gene encoding fumarase (EC. 4.2.1.2) is a rare cause of encephalomyopathy and a far more common cause of leiomyomas of the skin and uterus and of renal cancer [6]. TCAC dysfunction may also result from concurrent impairments in several steps of the cycle. For instance, combined deficiencies in SDH and aconitase (EC. 4.2.1.3) is observed in Friedreich's ataxia [7].

**Table 1:** Primary deficiencies in tricarboxylic acid cycle enzymes in humans.

Enzyme^1^	Clinical presentation	References
Fumarase	1. Progressive encephalopathy 2. Hereditary leiomyomatosis and renal cell cancer	1. [19] 2. [27]
Malate dehydrogenase	No disease identified so far	
Citrate synthase	No disease identified so far	
Aconitase	No disease identified so far	
Isocitrate dehydrogenase	Low-grade gliomas	[32]
α-ketoglutarate dehydrogenase	Congenital lactic acidosis	[28]
Succinyl CoA ligase	Encephalomyopathy with mtDNA depletion	[33]
Succinate dehydrogenase	1. Encephalopathy (Leigh syndrome) 2. Pheochromocytoma and paraganglioma	1. [20] 2. [34]

^1 ^Only primary enzyme deficiencies caused by known gene mutations are indicated, excluding secondary deficiencies that may be from genetic (e.g., Friedreich's ataxia with aconitase and succinate dehydrogenase deficiencies) or non genetic origin.

Residual activities associated with TCAC impairments in humans vary widely and may determine the magnitude of organic acid accumulation [8]. Organic acid accumulation has been proven instrumental in initiating tumor formation related to SDH or fumarase deficiency [9].

The ratios between TCAC enzymes are consistent for each mammalian tissues presumably reflecting their metabolic demand, as shown three decades ago in the seminal study by Pette and Hofer [10]. This echoes the occurrence of metabolons in the mitochondrial matrix [11-13], allowing for efficient channeling of substrates and co-factors through the Krebs cycle and related enzymes such as transaminase [14]. Consequently, in addition to the determination of residual absolute activities, estimation of ratios between enzyme activities is an effective means of detecting partial but potentially harmful deficiencies. When used to assess respiratory chain activities, this approach enabled the identification of several gene mutations, even in patients with partial respiratory chain deficiencies [15,16].

At present, TCAC enzyme activities are measured using a series of independent assays that are both laborious and time consuming. We therefore developed a limited set of assays allowing both measurement of all TCAC enzyme activities and detection of abnormalities in enzyme activity ratios. We used these assays successfully to detect severe and partial isolated deficiencies in several TCAC enzymes.

## Results

Given that TCAC enzyme activity ratios, because of their consistency [17], are important in comparing data between samples, we devised a method for measuring the activities of all eight TCAC enzymes using only three assays, which allows rapid determination of enzyme activity ratios. To define appropriate assay conditions, we first used mouse heart samples and assessed various parameters (detergent, pH, ionic force) that are known to independently stimulate each activity, but which might interfere with the measurement of other activities. We found that two media were sufficient for assaying all TCAC activities. The difference between these two media lies in the presence of phosphate required by some of the enzymes and in the use of electron acceptors to cope with the various reduced equivalents.

**Figure 1:**
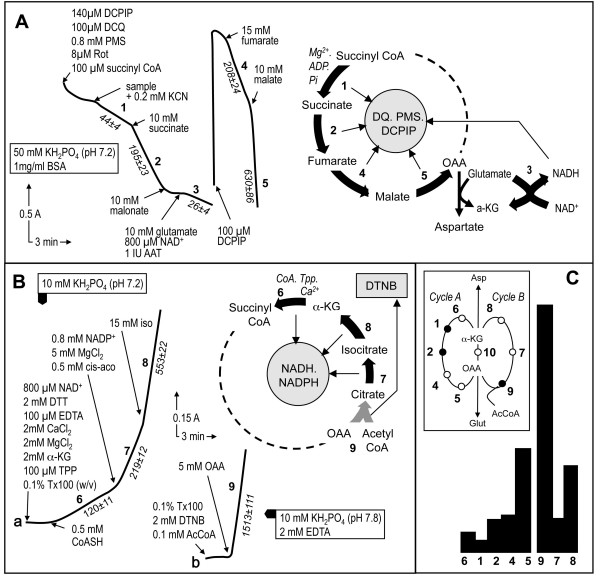
**Tricarboxylic acid cycle enzyme assays**. **A**, The first segment of the tricarboxylic acid cycle can be conveniently evaluated using a single assay to measure five enzymes by spectrophotometrically recording the reduction of DCPIP. The sequential assay begins by measurement of succinyl-CoA ligase activity based on oxidation of the produced succinate by succinate dehydrogenase, which forwards the electrons to the electron acceptors (DQ, DCPIP, and PMS). Coupling of these two activities to estimate succinyl-CoA ligase activity is permitted by the much higher activity of SDH than of succinyl-CoA ligase. After SDH inhibition by malonate, simultaneous addition of glutamate and aspartate aminotransferase ensures elimination of any oxaloacetate in the assay medium, thereby allowing further measurement of MDH activity. Incidentally, the required presence of NAD+ permits the measurement of glutamate dehydrogenase activity. Adding more DCPIP allows subsequent measurement of fumarase and MDH activity. Again, the coupling assay to estimate fumarase activity using MDH activity is permitted by the much higher activity of MDH. **B**, A second spectrophotometric assay subsequently measures pyridine dinucleotide reduction by three additional enzymes starting with α-ketoglutarate dehydrogenase. The next enzyme to be measured is aconitase, whose product, isocitrate, is readily oxidized by isocitrate dehydrogenase, producing NADPH. A saturating isocitrate concentration is finally added to enable measurement of isocitrate dehydrogenase activity. **C**, respective proportions of TCAC enzyme activities in mouse heart. The inset shows the TCAC depicted as two interacting enzyme cycles, A and B. Cycle A: α-ketoglutarate dehydrogenase (6), succinyl CoA ligase (1), succinate dehydrogenase (2), fumarase (4), and malate dehydrogenase (5); Cycle B: citrate synthase (9), aconitase (7), and isocitrate dehydrogenase (8). The two cycles interact via the activity of aspartate aminotransferase (10).

The first assay measures five enzymes sequentially in an individual sample (Fig. 1A). Importantly, while four of these enzymes catalyze steps of the TCAC, one, GDH, is measured as a consequence of the required presence of glutamate for the assay of MDH. Glutamate is required for the added aspartate amino transferase reaction in order to transaminate the oxaloacetate produced by MDH, which otherwise would rapidly block this last enzyme (Fig. 1B). The biological sample is first added to a detergent-containing medium allowing substrates and electron acceptors free access to their respective binding sites on the proteins. However, we found that succinyl-CoA batches variably contained reducing agents capable of interacting with the electron acceptor mixture used in the assay. Therefore, the assay is started only after most of this non-enzymatic reaction is completed. Then, biological sample is added to enable measurement of the first enzyme, GTP- and/or ATP-forming succinyl-CoA ligase, based on the amount of succinate formed by the enzyme. The succinate is then readily oxidized to fumarate by SDH concomitantly with ultimate reduction of DCPIP. In this assay, electrons from succinate are transferred by SDH to either phenazine methosulfate or decylubiquinone, both capable of reducing DCPIP. Maximal SDH activity is then measured by adding a large amount of succinate. Adding malonate, a competitive SDH inhibitor, essentially abolishes DCPIP reduction. Subsequent addition of glutamate, because of the presence of added NAD^+^, allows estimation of NAD^+^-dependent GDH activity. Depending on the enzyme activity levels in the sample, it may be necessary at this point to add more DCPIP before performing the next assays (fumarase and MDH activity). Fumarase is assayed by adding a large fumarate excess, which is readily converted to malate by fumarase, this latter acid being used up by MDH to produce NADH and oxaloacetate. Owing to the presence of added aspartate aminotransferase and glutamate, oxaloacetate does not accumulate and, therefore, does not slow the MDH reaction. The last enzyme of the assay, MDH, is then measured by adding 10 mM malate.

The second assay starts with measurement of the reduction of pyridine nucleotides (NAD^+^/NADP^+^) by KDH. This enzyme, one of the limiting steps of the TCAC, requires the presence of Ca^++ ^ions, thiamine pyrophosphate, and coenzyme A to catalyze the oxidation of α-ketoglutarate. After KDH measurement, cis-aconitate is added for measurement of aconitase activity based on the formation of isocitrate, which, in the presence of IDH, is readily used up to reduce NAD^+^/NADP^+^. Finally, the maximal activity rate of IDH is determined after addition of a large isocitrate excess. Citrate synthase, the last TCAC enzyme to be measured, condenses acetyl-CoA and oxaloacetate into citrate while concomitantly releasing coenzyme A, whose thiol residue readily reacts with Ellman's reagent (dithionitrobenzene). It is measured using the standard procedure which, in the case of cultured skin fibroblasts, concomitantly allows the detection of mycoplasma [18].

**Figure 2:**
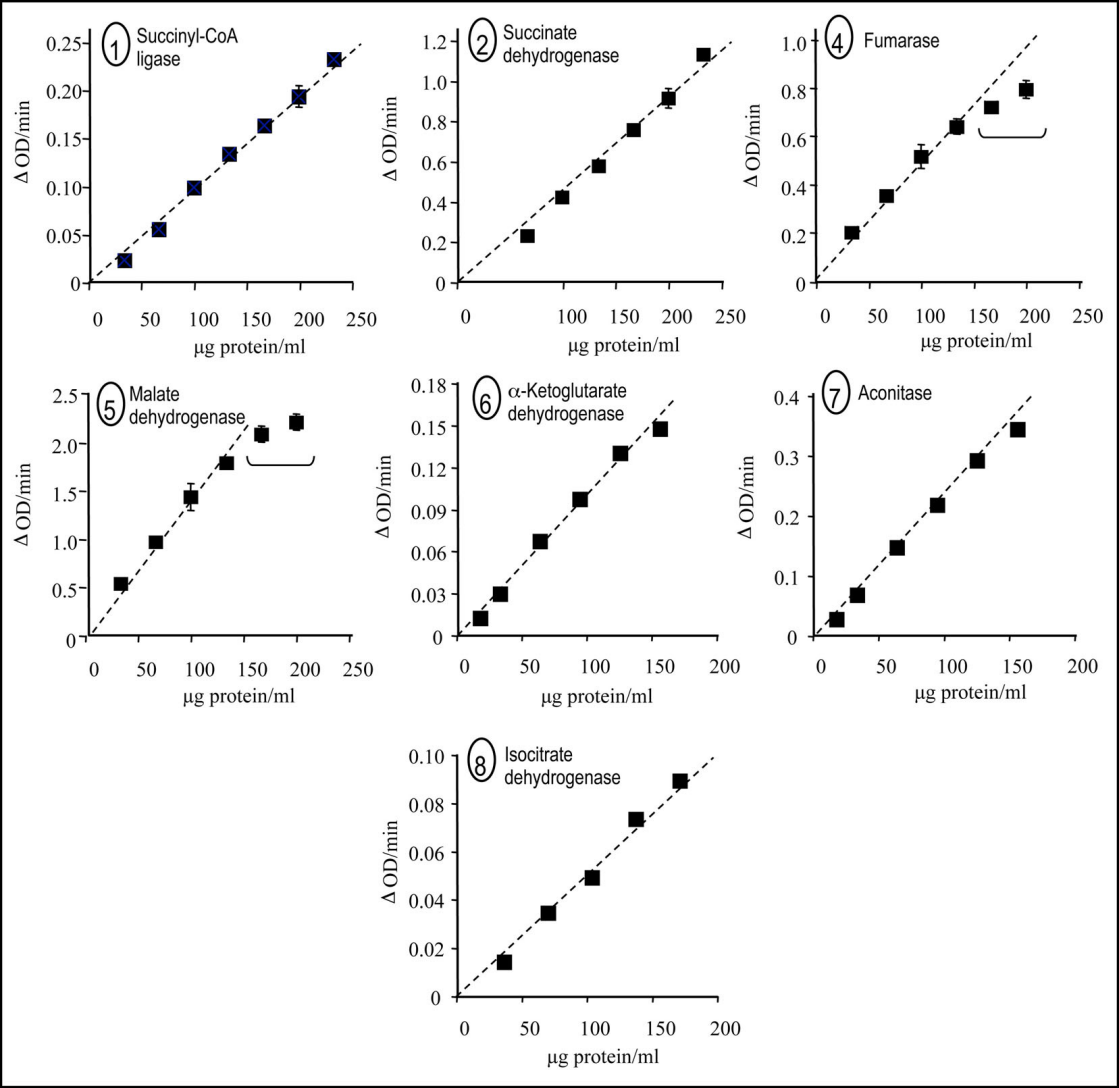
**Proportionality between TCAC enzyme activities and protein concentration in mouse heart homogenate**. 1-2, 4-8: Enzyme activities plotted as a function of protein concentration: succinyl-CoA ligase (1), succinate dehydrogenase (2), fumarase (4), malate dehydrogenase (5), α-ketoglutarate dehydrogenase (6), aconitase (7), and NADP^+^/NAD^+^-isocitrate dehydrogenase (8). Experimental conditions were as described in Figure 1. Enzyme numbering according to Figure 1.

Since part of these assays relies on coupling between several successive enzymes, e.g., aconitase and IDH, we evaluated the proportionality/linearity of these assays as a function of protein concentration in heart sample homogenate (Fig. 2). For protein concentrations of up to 150 μg per ml, each assay exhibited a linear response. Given that the protein concentration presumably depends on the extent of mitochondria enrichment in the tissue/cell under study, linearity should be evaluated before running quantitative assays on any tissue/cell.

**Figure 3:**
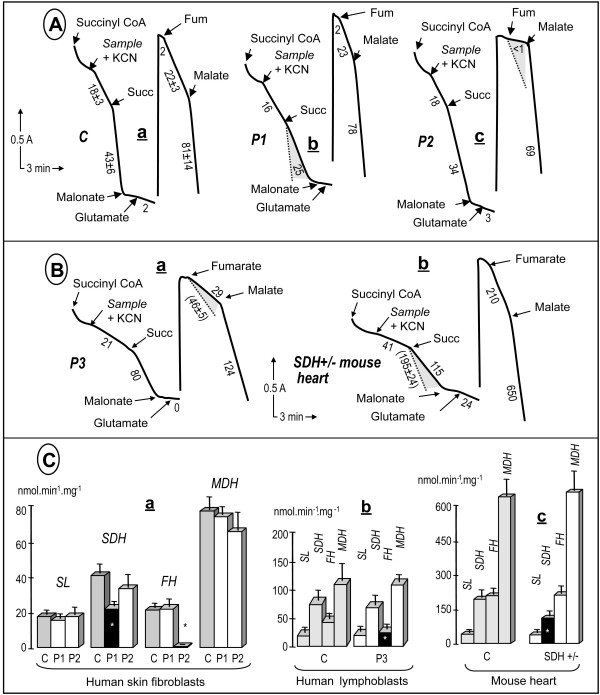
**Detection of severe and partial TCAC enzyme deficiencies in various biological samples**. A, severe enzyme deficiencies. (a) control fibroblasts; (b) SDHA-mutant fibroblasts homozygous for a deleterious R554W mutation, and (c) fumarase-mutant fibroblasts homozygous for a deleterious E319Q mutation. Note that only the addition of organic acids and inhibitors is indicated, although the experiments also involved additions of cations, cofactors, etc, similar to those in Figure 1. B, partial enzyme deficiencies. (a) fumarase-mutant lymphoblasts heterozygous for an N64T mutation; and (b) heart homogenate from a mutant mouse heterozygous for a deleterious SDHB deletion of exon 2. Numbers along the traces are nmol/min/mg protein. The shaded areas show the decreases compared to control values. C, Graphical representation of the values obtained for the various samples investigated. Dark symbols indicated statistically significant deficiencies. Values are means ± 1 SD. A minimal number of three independent assays (up to ten) were performed to calculate the mean values. Experimental conditions were as in Figure 1. Abbreviations: P1, patient harboring a homozygous SDHA mutation; P2, patient harboring a homozygous fumarase mutation; P3 patient harboring a heterozygous mutation; succ, succinate; fum, fumarate.

Finally, to evaluate the ability of our assays to detect deficiencies in specific TCAC enzymes, we investigated an array of samples with previously identified genetic defects resulting in deficiencies in various TCAC enzyme activities (Fig. 3). We first studied cultured human fibroblasts harboring mutations in either the *SDHA *or the *fumarase *gene (patients P1, P2; Fig. 3b, c). In agreement with our previous studies [19,20], we found that the *SDHA *mutation caused an about 60% decrease, whereas the *fumarase *gene mutation resulted in nearly total loss of fumarase activity. Interestingly, the loss of SDH activity did not hamper our ability to measure succinyl-CoA ligase activity, which was roughly similar to the control value. Then, we evaluated whether our TCAC assay was able to detect partial loss (about 50%) of fumarase activity. We studied a lymphoblastoid cell line from a human patient (P3) harboring a heterozygous mutation in the *fumarase *gene, previously shown to result in a nearly complete loss of activity when associated with a loss of the corresponding allele in tumors. Again, our assay proved capable of detecting the predictable partial (~40%) loss of fumarase activity in these cells, in terms of both the absolute activity and the activity relative to the other TCAC enzymes in the sample (Fig, 3d). Finally, heart samples from a mouse heterozygous for a deleterious mutation in the *SDHB *gene (exon 2 deletion) were investigated (Fig. 3e). We observed a consistent 40% decrease in SDH activity, as predicted by the heterozygous status of the animal.

## Discussion

The renewed interest in measuring TCAC enzyme activity, shown to be sensitive targets under various pathological conditions, prompted us to devise a rapid assay method for detecting TCAC deficiencies in biological samples. Our previous work on the mitochondrial respiratory chain established that, in addition to absolute residual activities, relative ratios of enzyme activities in a metabolic pathway are effective in detecting even partial deficiencies in a given enzyme. We therefore developed a set of three assays that conveniently estimate all TCAC enzyme activities in tissue homogenates and permeabilized cells. Although the experimental conditions had to be adapted to allow for the measurement of many enzymes using a small number of assays, they were largely based on the pioneer work done in the 1940s by Krebs and colleagues[21]. In particular, the concentrations of substrates and cofactors and the metal requirements for each enzyme were as determined by these authors.

As a first result of this work, we fully confirmed that TCAC enzyme activity ratios in each of the different tissues or cell investigated are consistent under basal conditions, as previously observed by Pette and colleagues as early as 1960[10,22].

To date there has been a lot of efforts to provide convenient assay procedures for respiratory chain enzymes [16,23-25]. In contrast, to our knowledge, there is no report on any convenient enzymatic procedure to measure the overall activity of TCAC enzymes in the context of screening procedures. Although our assays are rapid and sensitive, they have intrinsic limitations. First, three of the enzymes (succinyl-CoA ligase, fumarase, and aconitase) are measured via coupled assays involving the next enzyme in the cycle (SDH, MDH, and IDH, respectively). Obviously, a severe deficiency in the next enzyme would impair the ability of the assay to measure the first enzyme. Therefore, deficiencies in two consecutive enzymes should be evaluated by assaying each enzyme activity separately via standard methods. Second, although our assays are sufficiently sensitive to detect even partial deficiencies in one TCAC enzyme, measuring the slower enzymes via coupled assays (e.g., aconitase) requires a sample that is large enough to avoid problems with product dilution (e.g., isocitrate in the case of aconitase), which would impair the activity of the coupled enzyme (e.g., IDH in the case of aconitase). Despite these limitations, our set of assays enabled us to detect all TCAC enzyme deficiencies. Even a 40% decrease in fumarase activity in lymphoblastoid cell lines was readily detected.

So far there has been only a limited number of diseases which have been associated with primary isolated or multiple defect of the TCAC [7,19,20,26-28]. Beside primary defects of the TCAC genes, as some of the TCAC proteins harbor oxygen-sensitive iron-sulfur cluster, *i.e*. aconitase, or require a full set of co-factors, i.e. α-ketoglutarate dehydrogenase, a loss of activity - secondary but yet possibly instrumental in the pathophysiological process - might well be observed in a number of conditions such as aging, Parkinson's disease or heart failure.

## Methods

### Biological samples

Fibroblasts derived from forearm biopsies taken with informed consent from healthy controls and patients with TCAC enzyme deficiencies were grown under standard conditions as described elsewhere [29] and frozen (-80°C). Before use, cells were resuspended in 1 ml of medium (A) composed of 0.25 M sucrose, 20 mM Tris (pH 7.2), 40 mM KCl, 2 mM ethylene glycol tetra acetic acid (EGTA), 1 mg/ml bovine serum albumin (BSA), 0.01% digitonin (w/v), and 10% Percoll (v/v). After 10 min incubation at ice-melting temperature, the cells were centrifuged (5 min × 2,300 *g*), the supernatant discarded, and the pellet washed (5 min × 6000 *g*) with 1 ml of medium A devoid of digitonin and Percoll [30]. Lymphoblasts from patients harboring a deleterious heterozygous fumarate hydratase gene mutation (N64T) were processed similarly to the cultured fibroblasts. Mouse colony was maintained in accordance with national and institutional guidelines. Animal procedures were approved by the ethical review panel of the Robert Debré Institut, Paris, France. Hearts were obtained from mice, snap frozen in liquid nitrogen and stored at -80°C. Frozen tissues were homogenized at ice-melting temperature by hand using a glass-glass potter in medium (1/10-1/20 v/v) composed of 20 mM Tris (pH 7.2), 0.8 M sucrose, 40 mM KCl, 2 mM EGTA, and 1 mg/ml BSA. Large cell debris was removed by low-speed centrifugation (1,500 *g *for 5 min).

### Spectrophotometry

The first assay measures succinyl-CoA ligase, SDH, glutamate dehydrogenase (GDH), fumarase, and malate dehydrogenase (MDH) (see below; Fig. 1). This assay is performed in 400 μl of medium A containing 50 mM KH_2_PO_4 _(pH 7.2) and 1 mg/ml BSA. The reduction of dichlorophenol indophenol (DCPIP) is measured using two wavelengths (600 nm and 750 nm) with various substrates and the electron acceptors decylubiquinone and phenazine methosulfate. The second assay measures α-ketoglutarate dehydrogenase (KDH), aconitase, and isocitrate dehydrogenase (IDG) activities. The same volume of the same medium is used, and pyridine nucleotide (NAD^+^/NADP^+^) reduction is measured with various substrates using wavelengths of 340 nm and 380 nm. In the third assay, citrate synthase is measured by monitoring dithionitrobenzene (DTNB; Ellman's reagent) reduction at wavelengths of 412 nm and 600 nm as previously described[19]. For this study, all measurements were carried out using a Cary 50 spectrophotometer (Varian Inc., Palo Alto, CA) equipped with an 18-cell holder maintained at 37°C. Protein was measured according to Bradford [31]. All chemicals were of the highest grade from Sigma Chemical Company (St Louis, MO).

## List of abbreviations

AAT: Aspartate Aminotransferase; AcCoA: AcetylCoA; Asp: aspartate; BSA: Bovine Serum Albumin; cis-aco: cis-aconitate; DCPIP: Dichlorophenol Indophenol; DQ: Duroquinone; DTNB: Dithionitrobenzene; DTT: Dithiothreitol; EDTA: Ethylene Diamine Tetraacetic Acid; Glut: glutamate; GDH: Glutamate Dehydrogenase; IDH: Isocitrate Dehydrogenase; iso: isocitrate; KDH: α-Ketoglutarate Dehydrogenase; α-KG: α-ketoglutarate; MDH: Malate Dehydrogenase; OAA: oxaloacetate; PMS: Phenazine Methosulfate; Rot: rotenone; SDH: Succinate Dehydrogenase; TCAC: Tricarboxylic Acid Cycle (Krebs cycle); TPP: thiamine pyrophosphate; Tx100: Triton X100.

## Authors' contributions

SG, JJB and PR designed the concept and experiments of the study. VP, ED and PB provided and studied the various cell types utilized, while JF and APGR provided and studied the SDH-mutant mouse. SG and PR analyzed the data. PR drafted the manuscript, helped by the other authors. All authors approved the final manuscript.
